# Losing Life's Sparkle: Experiences of Canadian Choral Musicians During the COVID-19 Pandemic

**DOI:** 10.1177/00084174221145823

**Published:** 2023-01-22

**Authors:** Mary Jo A. Lozano, Stephanie L. Churcher, Madison J. Kirchner, Teri M. Slade

**Keywords:** music, qualitative research, occupational disruption, occupational alienation, Aliénation occupationnelle, disruption occupationnelle, musique, recherche qualitative

## Abstract

**Background.** Singing in choirs, which previous research has identified as supporting wellbeing, has been restricted and altered during the COVID-19 pandemic. **Purpose.** The purpose of this study is to investigate and describe the experience of music-making for musicians in professional and semi-professional choirs in Canada 18–22 months into the COVID-19 pandemic. **Method.** Semi-structured interviews were conducted with 11 participants and analyzed using interpretive description. **Findings.** Four themes: (1) increased negative feelings associated with the music-making experience due to COVID-19 restrictions, (2) isolation and disconnection, (3) recognizing how music-making aids in their own mental health, the participants used music-making to help their communities cope with the pandemic, and (4) adapting in response to COVID-19 reinforced music-making's importance. **Implications.** Understanding how the COVID-19 pandemic has altered Canadian choral musicians’ experience of music-making can help occupational therapists in supporting choral musicians return to this meaningful occupation.

## Résumé

**Description.** La pratique du chant choral, identifiée par les chercheurs comme étant favorable au bien-être, a été restreinte et al.térée durant la pandémie de COVID-19. **But.** Le but de cette étude est d’examiner et de décrire l’expérience de la pratique musicale chez des musiciens de chorales professionnelles et semi-professionnelles au Canada entre 18 et 22 mois après le début de la pandémie de COVID-19. **Méthodologie.** Des entrevues semi-structurées ont été réalisées auprès de 11 participants et analysées à l’aide d’une description interprétative. **Résultats.** Quatre thèmes ressortent : 1) L’augmentation des sentiments négatifs associés à l’expérience de la pratique musicale en raison des restrictions liées à la COVID-19; 2) L’isolement et la déconnexion; 3) Reconnaissant que la pratique musicale était utile à leur santé mentale, les participants ont utilisé la musique pour aider leurs communautés à passer à travers la pandémie; 4) L’adaptation en réponse à la COVID-19 a renforcé l’importance de la pratique musicale. **Conséquences.** Le fait de comprendre comment la pandémie de COVID-19 a altéré l’expérience de la pratique musicale pour les musiciens de chorale peut aider les ergothérapeutes à soutenir les musiciens dans leur retour à l’exercice de cette activité significative.

## Introduction

The COVID-19 pandemic has posed challenges for choral musicians, who typically need to be in close physical proximity with each other to practice, and to have the presence of a live audience for performances. This paper explores the experiences of high-level choral musicians in Canada during this time of adversity, in order for occupational therapists to be aware of the challenges and strengths of these individuals. For the purpose of this paper, high-level choral musicians refer to those who contribute musically to professional or semi-professional choirs, that is, choirs that are routinely or occasionally paid for their music-making activities. High-level choral musicians engage in music-making with choirs at a professional skill level, even if they themselves do not receive payment. The term choral musicians includes singers, directors, and collaborative pianists. It is important for this population to have professional and psychological support during the pandemic, and through the COVID-19 pandemic's emotional and physiological consequences that will continue to surface in months and years to come (Primov-Fever et al., 2020).

## Background

Choir singing is enjoyed by many and is beloved for the sense of purpose, social connectedness, and mental and physical wellbeing it provides (Clift et al., 2010; Dingle et al., 2013; Grunwald Associates & Chorus America, 2019). Multiple qualitative studies have found that participants experience improvements in their mental wellbeing, cognition, and sense of connectedness after choir practice and performance (Bailey & Davidson, 2005; Damsgaard & Brinkmann, 2021; Pérez-Aldeguer & Leganés, 2014; Perkins et al., 2020).

Despite the reported benefits of music-making, professional musicians do not always experience the same levels of wellbeing as amateur musicians (Bonde et al., 2018). Occupational health concerns of professional singers include muscle tension, hearing problems, high production of stress hormones, a higher prevalence of anxiety than the average population, and an above average intake of alcohol and drugs (Bonde et al., 2018; Hildebrandt et al., 2012; Isaac et al., 2017). Musicians in Canada are often self-employed, with minimal additional health coverage, insurance, or employee assistance (Neil, 2010). Viruses, gastroesophageal reflux, and vocal fold nodules are all issues that may render singers unable to use their voice for a time (Guptill, 2014). Importantly, the singer's voice becomes a part of their identity (Turpin, 2022), meaning that when faced with a health issue where their voice is vulnerable to dysfunction, the overall emotional and mental wellbeing of the individual can be impacted (Rohrer, 2017). Though professional musicianship and choral singing comes with risks, choral musicians love what they do, and choirs of all skill levels have been described as places of “artistic creation, wellbeing and community care” (Youngblood et al., 2021). Occupational therapists have contributed to the care of musicians by advocating for more affordable care, public policy development, research, and mental health care among other roles (Guptill, 2014).

In March 2020, choral singing began to be considered dangerous in light of the risk of coronavirus transmission, despite its longstanding association with singer wellbeing. The beginning of the COVID-19 pandemic in many choir communities was marked by “super spreader” events in choirs such as the Skagit Valley Chorale in Washington State, the Amsterdam Mixed Choir, the Berlin Cathedral Choir, and the Voices of Yorkshire choir in England (McKie, 2020). Initial studies attributed these events to the projection of one's voice and resultant increased production of droplets and aerosols (Alsved et al., 2020; Good et al., 2021; Stockman et al., 2021). Though recommendations would later be developed to mitigate risks, in-person choral singing became impossible in many contexts due to the risk of aerosol spread, close proximity of choral musicians, the recent memories of super-spreader events, and the introduction of public health restrictions (Stockman et al., 2021; Youngblood et al., 2021).

Choirs adapted quickly, with many moving to online rehearsals. Research focused in the United States, the United Kingdom, and Israel noted mixed results and satisfaction with this change. Youngblood et al. (2021) surveyed and interviewed professional and non-professional musicians in the United States who reported finding online music-making “less satisfying” or even “exhausting and boring.” Some participants decided not to engage in online rehearsals completely until it was safe to rehearse in-person again, while others found that without a performance to prepare for, they could not find the motivation to practice. A survey conducted by Spiro et al. (2021) found that 85% of musicians and performers felt more anxious at the beginning of COVID-19 lockdowns, which some attributed to the future of their finances and future career, as well as the wellbeing of their family and friends.

With the experience of increased anxiety during the COVID-19 pandemic, professional musicians experienced risks in addition to those associated with contracting COVID-19. Primov-Fever et al. (2020) investigated the experience of professional singers and actors during the first months of the COVID-19 pandemic using the Generalized Anxiety Disorder-7 questionnaire and a “Voice COVID-19 Questionnaire.” Many singers experienced a heightened sense of anxiety at the possibility of harming their own or others’ health through their music-making and recorded significantly higher stress levels than the general population at the time. Some singers experienced “vocal avoidance” and heightened self-awareness and concern about their voice. This is a concern, as choral musicians singing under stress over a long period of time are vulnerable to developing acute or chronic voice disorders (McBroom, 2017). Undoubtedly, the COVID-19 pandemic has created a unique set of challenges to the occupational engagement of choral musicians.

Occupational therapists are well-positioned to assist choral musicians in the recovery process for challenges associated with the COVID-19 pandemic. Many care providers lack knowledge regarding the health of professional singers (McKinnon-Howe & Dowdall, 2018). Occupational therapists can provide rehabilitation, psychotherapy, and support musicians to access appropriate benefits and social services; they can work in public policy development to support musicians’ health, and analyze the physical, mental, and emotional demands of choir (Guptill, 2014). When working in public policy development, occupational therapists embrace concepts of occupational justice to combat experiences of occupational deprivation, disruption, and alienation among others (Whiteford, 2000). Opposing these occupational injustices create a society where people have the freedom and opportunity to choose and engage in occupations that are useful and meaningful in their environment (Whiteford, 2000). As we begin to address the physical and psychological consequences of the COVID-19 pandemic and associated public health restrictions, it is important that occupational therapists and other care providers are aware of the environment and experiences of individuals that they may provide care for.

## Purpose

While several studies have articulated the experience of musicians across genres at the beginning of the COVID-19 pandemic, the continued experience of high-level choral musicians in the second year of the pandemic has, to the authors’ knowledge, not been investigated. The purpose of this research project is to investigate the experiences of choral musicians in Canada during the COVID-19 pandemic as they engaged in music-making, in order for occupational therapists to better understand elements that contribute to the occupational wellness or illness of musicians.

## Research Question

*How do high-level choral musicians experience the occupation of music-making 18–22 months into the COVID-19 pandemic?* In this paper, occupation is defined as “all manner of human doing,” including paid or unpaid employment, daily self-care activities, household management, and leisure activities (Townsend & Polatajko, 2013, p. 17).

## Methods

### Participant Recruitment & Data Collection

Semi-structured interviews were conducted one-to-one over Zoom with 11 high-level choral musicians, defined as singers, directors, composers, and conductors who engage in music-making with professional and/or semi-professional choirs, from across Canada (see Table 1 for brief descriptions of participants). A Canada-wide email newsletter for choral musicians was used to recruit participants. Interested ensemble directors also distributed recruitment emails to choir members and professional connections. This represents both purposive and snowball sampling, allowing us to include both high-level choral musicians who are engaged in the national choral scene and those who are not. The last listed author conducted open-ended interviews with each participant. The interview guide included questions about the participants’ music-making experiences before and during the COVID-19 pandemic, the effects of the pandemic on their music-making engagement, what meaningful music-making means to them, and whether they had been able to engage in meaningful music-making since the beginning of the COVID-19 pandemic (see Supplemental Materials for interview guide). Interviews were transcribed verbatim and analyzed in the qualitative data analysis software program Quirkos. ML, SC, and MK collaboratively analyzed the data. Quirkos was chosen to assist with analysis because of its interactive environment for multiple users to collaborate during data analysis. Ethics approval for this project was obtained from the Research Ethics Office of the University of Alberta.

**Table 1:** Participant Characteristics

Participant pseudonym	Age range	Geographical region	Involvement in music community
Aidan	20–29	Ontario/ Quebec	Amateur, tenor; sings in a chamber choir
AmyDee	30–39	Ontario/ Quebec	Semi-professional, alto/mezzo-soprano; sings in a chamber choir, occasional paying gigs; administrator
Henry	50–59	Ontario/ Quebec	Semi-professional, bass; multiple ensembles including a touring project choir
Joy	40–49	Western Canada	Professional, alto; sings in a professional project choir; director of a children's choir
Jonathan	30–39	Ontario/ Quebec	Amateur, baritone; sings in a semi-professional choir and in musical theatre; administrator
Kayleigh	20–29	Atlantic Canada	Amateur, alto; sings in a project choir; diverse musical background; university student
Maria	30–39	Western Canada	Professional, alto; sings in a professional project choir; choir conductor; composer; music teacher
Madeline	30–39	Ontario/ Quebec	Amateur, alto; sings in a high-level community choir
Norah	30–39	Atlantic Canada	Professional, mezzo soprano/contralto; choir director; teacher; performer
Rochelle	30–39	Atlantic Canada	Semi-professional, soprano; multiple ensembles
Simon	30–39	Ontario/ Quebec	Professional, ensemble director/conductor; church music director

### Data Analysis and Interpretation

This project used a phenomenological approach to interpretive description. Interpretive description is a method for qualitative analysis that is commonly used in health-related disciplines for its theoretical integrity and applicability to real-world practice (Thorne, 2016). Interpretive description invites the researcher to integrate knowledge and clinical reasoning from their practice discipline into their interpretation and description of qualitative data, and does not dictate a formal methodology for data analysis. The purpose of interpretive description is to generate new inquiry and insight that leads to deeper understanding of what could be an optimal response in clinical practice. Thorne (2016) writes that clinically-oriented research should be informed by patients’ lived experience, since interactions between healthcare professionals and patients are so intimate and individual. Using a phenomenological approach to interpretive description emphasizes the lived experience of the people served by clinicians, allowing the evidence that guides clinical practice to be more deeply client-centered. Studies done by Shalanski and Ewashen (2019) and Li et al. (2021) followed a similar methodology in bringing phenomenological paradigms to interpretive description research projects.

The findings of a phenomenological study are a compilation of descriptions of the meanings of lived experiences of individuals (Sloan & Bowe, 2014). Hermeneutics, the interpretation of language by an observer, reflects on the content of spoken or written accounts of lived experiences to derive something “meaningful” and “thematic” about that experience (Sloan & Bowe, 2014, p. 4). The assertion that it is impossible for an observer to remove themselves from the process of “essence-identification” led the authors to identify hermeneutic phenomenology as an appropriate branch of interpretive description for this project. Hermeneutic phenomenology acknowledges that the researcher cannot remove themselves from the process of identifying the essences of participant experience. However, through the process of reflexivity, the researcher can both practice critical self-awareness of their assumptions, while making use of relevant prior experience and empathy for the participants to aid analysis and interpretation of the meanings of a text (van Manen & Adams, 2010; Sloan & Bowe, 2014).

This research methodology requires the authors to first “get to know the text” (Sloan & Bowe, 2014, p. 12), and engage with the parts of the information and the whole in a dialogic manner (van Manen & Adams, 2010). The authors ML, SC, and MK read through the interview transcripts individually while reflecting on what the overall themes and meanings of each interview might be. The authors used Quirkos software to inductively code interviews, highlighting text that spoke to the essence of each participant's experience. The authors reflected individually and collaboratively on possible themes within the transcripts, while also considering ways the interview questions, methods, and researcher positionality were related to the themes constructed.

After reading most of the transcripts, authors collaboratively organized the codes into themes, common to several or all of the participants, in the map of visible themes in the Quirkos software. All of the authors met to reflexively discuss how the themes that occurred in the transcripts were similar to elements of their own experience or experiences of other musicians during the COVID-19 pandemic, and critically considered how the experiences were different and individual to the participants’ experiences. The themes that the authors developed in initial readings of the data were used to inform second, third, and fourth readings of the transcripts, in which authors ML, SC, and MK interpreted the ways in which the data could respond to the research question. Themes were revised through this process to ensure the participants’ experiences were portrayed with fidelity and clarity.

### Researcher Positionality

Interviews were conducted by author TS, a research associate in occupational therapy who is actively engaged in the Canadian music community as a pianist and vocalist. TS is a member of the national choir community. The shared knowledge of Canadian choir culture supported rapport and candidness in the interview setting. Recruitment materials made clear who would be conducting the interview so that potential participants who were acquainted with the interviewer could make an informed decision about their participation. This may have excluded potential participants who would have felt uncomfortable speaking about their experiences with this author. TS is not a director, manager, or otherwise in a position of power in the Canadian music community. TS did not participate directly in data analysis, which allowed ML, SC, and MK to analyze the data with an outsider's perspective. The combination of insider and outsider perspectives on the research team promoted a rich interactive analysis of the data. Analysis was conducted by authors ML, SC, and MK, three Canadian, cisgender female occupational therapy students who enjoy listening to music, attending live performances, and engage occasionally in amateur music-making activities or music-making as a dimension of their spirituality. These three authors recognize that their background in occupational therapy, which emphasizes the impact of engagement in meaningful occupations on overall health and wellbeing, influenced the research process, particularly their interpretation of the experiences and meaning expressed in the interview data. Furthermore, all authors experienced other forms of disrupted occupational engagement in their personal lives as a result of the pandemic, which situates the research within a shared lived experience between researchers and participants. TS reviewed the findings for community relevance. Group meetings were conducted throughout the data analysis process to facilitate researcher reflexivity. These meetings provided a space for the authors to reflect on personal biases and discuss rationale behind decisions made during data analysis.

## Results

### Theme 1: Increased Negative Feelings Associated With the Music-Making Experience due to COVID-19 Restrictions

**Feelings of fear and anxiety.** Participants described negative feelings, like fear and anxiety, became a more profound part of the music-making experience after multiple news stories and webinars announced choral singing events were associated with the spread of COVID-19. Aidan could “pinpoint to the hour, the worst day of the entire pandemic and it was the day that the…ACDA [American Choral Directors Association] did that big webinar about COVID-19 and aerosol spread and singing.” Henry found “it was quite distressing to learn that singing was causing people to die.” From then on, participants discussed having an added layer of anxiety with regard to their choral singing. As Kayleigh put it, “you couldn't see people, you could not talk to people, but singing…specifically in choirs, was 100 times worse…there's just so many nerves about it.” Madeleine was “the most anxious [she has] ever been.” Rochelle worried about what COVID-19 “has done to choral singing both in vilifying the singing itself and also what choral singing can be right now, and I worry the effect that [COVID-19] will have long term.”

**More time worrying, less time music-making.** With the concern that singing in choir was unsafe, participants described being vigilant with following lockdown restrictions. Consequently, participants spent less time engaging with music-making due to the increased time spent keeping up with public health restrictions. Norah found “it takes so much more time away from [the music] you would have been preparing for the actual rehearsal.” Figuring out “what's legal for us to do and not to do” (Jonathan) and implementing new procedures to “set up differently… do temperature checks … figure out the distancing” (Aidan) were added steps to the routine for choir directors and board members in particular. With the need to balance public health restrictions, participants found it “hard to make the music the primary focus” (Maria).

**Positive aspects of music-making reduced.** When participants were able to spend time in rehearsals, restrictions took away the positive aspects of choir that participants normally experienced when singing in choir. For some ensembles, this involved wearing face masks, distancing singers from one another during rehearsal, and/or meeting to sing in much smaller groups than usual. Participants were “surprised at just how much of a barrier the masks and the distancing [are]” (Rochelle). Joy, among others, found it “hard to sing in a mask.” The “muffling of those cloth masks was a real problem… everything was quiet” (Simon). Upon returning to performing, Maria was “expecting to have that…’oh, I’m in heaven; I’m singing in a choir’ feeling and it didn't really come,” and said it was “because of the masks.” The “two meter distance actually made a big difference with listening to each other and blending” (AmyDee). There was not a “sense of ensemble necessarily anymore, just because you can't be close” (Joy). Participants discussed how the skill and capacity of a choir are limited by the restrictions. Rochelle suspects “there's a plateau past which we will not be able to proceed, because [the masks] limit…what we can do together, whether in rehearsal or in performance.” While participants were amenable to following these guidelines for the safety of themselves and others, the restrictions placed on choral singing diminished the positive features of the choir experience.

### Theme 2: Isolation and Disconnection

Participants recounted the impact that changes to their music-making had on their sense of connection to others. Elements of choral singing that were vital to making participants feel connected to the audience, connected to fellow choral musicians, and a sense of community, became disrupted, resulting in heightened feelings of isolation and disconnection.

**Isolation from the audience.** Some participants gave online performances, where the audience was physically absent from the performance space, and others gave in-person performances with audience members further distanced from performers and wearing masks. Rochelle described feeling “disconnected and removed from the audience.” She explained that the distance between herself and the audience made it difficult to spot in a crowd “someone with that stupefied look… having a spiritual moment that you helped create.” Many participants also reflected on the energy that they previously had drawn from audience feedback, which became unavailable when the audience was absent or distanced. Madeline discussed receiving “a particular charge” and “positive pressure” when there is an audience. Simon described the reduced performance of the choir at rehearsals, but not worrying because “as soon as the audience comes, [the choir is] going to be fine and that's what happened…they sang beautifully.”

**Isolation from fellow choir members in the rehearsal/performance space.** Participants discussed how challenging and unsatisfying in-person interactions with fellow choral musicians were, which made the participants feel disconnected. Jonathan described wanting to “smile and wave at [others],” which is difficult when everyone is physically distant. Joy mourned the experience of bantering with those sitting near her, as COVID-19 restrictions have other choral musicians sitting “too far away, so [she feels] very isolated.” Without physical touch, certain participants felt they could not fully interact with and respond to their peers; AmyDee explained that she bonds with others through physical touch, whether that be a hug, pat on the arm, or a touch on the shoulder if someone is upset. The presence of masks highlighted the necessity of seeing facial expressions for human interactions. Aidan expressed that conversing with masks on “can still be very gratifying and enjoyable but… there's clearly a step that's missing.” Maria described momentarily removing masks while physically distanced outdoors during a break, explaining that she felt “a change in how connected [she] felt to everybody just because [she could] see their face.” Rochelle identified that facial expression was instrumental to the satisfaction of the shared experience of choir, explaining, “we’re feeding off of seeing each other's expression… but without the facial expressions and the body language, we’re very limited.”

**Struggle to maintain a sense of community.** Participants discussed how the lack of in-person interactions with fellow choral musicians made it challenging to build or maintain the community bond that is vital to the choral singing experience. Participants described having a strong social connection to other members of their choir due to their shared values and outlook on music, which cannot be formed with others who are not part of high-level choirs. Kayleigh stated that choir is “the only time when I can…really connect to people who…feel the same kind of resonance with music.” AmyDee attributed the unique connection to her fellow choral musicians as “seeing people that care about choir and music making as much as I do.” As the pandemic unfolded and singing together became less accessible, what remained was one of the most integral elements of choir—social connection. In Aidan's experience, online choir consisted of “non-rehearsal things just to sort of keep the community alive.” With many choral musicians in his ensemble spending their days online for work, Simon explained that most musicians he knew “didn't have any appetite to be doing virtual choirs… so it became more about keeping the community engaged and connected.” When stripped of the ability to sing together, choral musicians still valued maintaining meaningful social bonds with one another—connections that are unique to these choir spaces. When the sense of community is disrupted, it contributes to an overall feeling of reduced connection.

### Theme 3: Recognizing How Music-Making Aids in Their Own Mental Health, the Participants Used Music-Making to Help Their Communities Cope With the Pandemic

**Music-making as a personal coping strategy.** Many participants identified engaging in choir as a way to cope with everyday troubles prior to the COVID-19 pandemic. Jonathan described choral singing as “one of [his] supports that helps [him] through difficult times,” while Rochelle discussed how choral singing is “a big part of what gives meaning and how we process emotions.” Madeline stated, “producing music with other people is something that feels profoundly human,” and describes engaging in choir music as “taking care of [one's] own spirit and supporting the people around you.” Others noted how choral singing is essential to mental health and wellbeing. AmyDee explained that choral singing is “one of the times where my head is at its clearest and… it's a huge part of my mental health.” Describing singing as something that improves his mental state, Henry explains it is “not just a source of creativity, but uh, also it's lifegiving, I think, and life-sustaining, and life reanimating, rejuvenating.”

**Using music-making to help others deal with the difficult reality of the pandemic.** Rochelle observed that “the collective trauma [of COVID-19] precipitated a desire and a hunger for people to connect to art, whether that was sculpture or painting, or…visual arts or music.” Recognizing the necessity of art as a way to help the community cope, the participants felt drawn to give back to the community, despite the challenges. Henry explained persisting in choral performances even if masking and distancing made it difficult because it was “to help the community rehabilitate… because the community was suffering.” Others described using alternative forms of music-making to help others cope, like Rochelle who shared daily music covers with her friends on social media in hopes that the songs “would give that kind of [emotional] outlet for others,” which she compares to listening to one's favorite album to emotionally process a breakup.

### Theme 4: Adapting in Response to COVID-19 Reinforced the Importance of Music-Making

**Determination to adapt.** Recognizing the significance of music-making in their lives, the participants described persisting through the challenges of adapting their music-making in context of the pandemic. For instance, Henry described adapting to the challenge of singing with a mask because “knowing the alternative, which is returning to lockdown and not singing, [is] what motivated us” (Henry). Others adapted by engaging in alternative forms of music-making because they had to “make [music] happen, because I just have to make it happen” (Joy), even if it “wasn't gonna be able to scratch that itch the same way” (Rochelle). For instance, participants engaged in new opportunities they “never would have done before… I was asked to be the adjudicator… for an online digital thing” (Norah about a festival). Another participant, Rochelle, started performing song covers and sharing it with friends on social media during the pandemic. Through this Rochelle realized her solo singing provided a “stark contrast” to choral singing, explaining that “elements [of choir] were missing and they can't be gotten elsewhere.”

**Importance of music-making reinforced.** Even as participants strove to maintain engagement in music-making throughout the COVID-19 pandemic, the loss of choral singing as it was prior to the pandemic reinforced its importance to the participants. Aidan loves “the music and the feeling of singing collectively and the emotional experience of it.” Norah believes that the act of music-making “does feed [her] soul and [her] heart and [her] mind.” COVID-19 changed the experience of music-making, and choral musicians such as Rochelle felt that “COVID-19 really crystallized what music is, for me, what I want from it, how I want to share it with whom I want.” Without music, Amy Dee had “an identity crisis, so throughout [the COVID-19 pandemic] I definitely felt out of sorts.” The loss of choir for Jonathan meant “that shimmer, that sparkle that is in life that I kind of aim to have some amount of in my world … was very much absent.” Madeleine found the early parts of the COVID-19 pandemic and public health restrictions an “unmooring experience… not having singing with other people to look forward to” and had difficulty visualizing how “whatever [she] was preparing and working on was going to fit into some larger whole.” These were all revelations that participants described learning because of the restrictions placed on choral singing due to the COVID-19 pandemic.

**Choral singing feels like home:** Among these high-level musicians, choir is more than just the physical act of singing. The importance of the participants’ experience of choir lies within the sense of comfort, social connectedness, and belonging that it provides, all of which were diminished with the onset of the COVID-19 pandemic and subsequent public health measures. Musicians coming back to sing together again in-person as a choral ensemble was a powerful moment after months of lockdowns, restrictions, and isolation. Though participants had to deal with difficulties of navigating choral singing with masking and/or distancing as discussed in theme 2, reunting with some semblance of in-person choir provided a sense of comfort. Participants’ descriptions of their return to choral singing highlight how it provides a sense of ease and relief to them, which is important when the stresses of life arise as it did during the pandemic. The return to a place where they belong when “so often it's really hard to find a place to belong in today's world” (Joy), was expected to be met with tears by Rochelle but instead, she expressed, “I was just too happy to cry, like I was just so … it just felt like going home.” (Rochelle).

## Discussion

The findings of this study detail how the experience of music-making for high-level choral musicians in Canada changed during the COVID-19 pandemic. Fear and anxiety became a significant feature of their experience. As the activity of choral singing was deemed dangerous in the context of the COVID-19 pandemic, participants often contended with discerning whether their participation in choir was “legal.” Other countries around the world also experienced this uncertainty; in Australia, choirs were reluctant to return unless the lifting of restrictions specifically mentioned choir (Salleh, 2021). The implementation of masks and distancing, while creating a sense of safety for participants, also resulted in a less satisfying experience of choir. Beyond these measures being a physical barrier, it obstructed participants’ ability to fully connect with fellow choir members and the audience. This experience reinforced that connection with others is a paramount aspect of the choir experience. Despite the challenges and dissatisfaction with music-making during COVID-19, participants still turned to music-making to cope during this time of adversity. Its use as a coping strategy by participants exhibits how music-making contributes significantly to their wellbeing. The threat of losing choral singing as they knew it reinforced for each of them its importance. Participants described choir as a piece of their identity, what feeds their soul and what adds sparkle to their life. Reuniting with this experience of in-person singing after months of lockdown provided many with a sense of comfort, belonging, and connectedness, even when these same choral singing experiences were altered by pandemic-related restrictions.

The experience of participants feeling anxious and disconnected from music-making aligns with research conducted earlier in the pandemic. During the beginning stages of the pandemic, musicians in various studies expressed how lockdowns limited their engagement in music; some were even reluctant to take part in music at home alone (Spiro et al., 2021). Many individuals “eulogized” their experience of music-making in person and professionally early in the pandemic; they had lost a source of income as well as the ability to rehearse and perform with their colleagues, without an anticipated return to their previous activities (Messick, 2021; Spiro et al., 2021; Youngblood et al., 2021). This temporary loss of music-making may be conceptualized by occupational therapists and scientists as occupational disruption. This is when a person's normal pattern of occupational engagement is disrupted temporarily due to changes in life or the environment (Whiteford, 2000). Lockdowns may have been temporary, but participants in the present study and others (Spiro et al., 2021; Youngblood et al., 2021) described fearing for the future and sustainability of music performance. This suggests that participants felt as though they were under the threat of occupational deprivation, a term used to describe a state in which people are permanently unable to do what is necessary or meaningful in their lives due to external restrictions (Whiteford, 2000).

Throughout interviews in the present study, participants described their experiences of music-making within the COVID-19 pandemic as dissatisfying. Similar studies described online efforts to engage in music as “better than nothing,” but also nowhere near the experience of the choir they knew and to which they longed to return (Youngblood et al., 2021). Cai et al. (2021) found that musicians lost the energy and motivation to engage in practicing music due to pandemic-induced stress. By the time of the interviews in the present study, participants had experienced some manner of returning to making music in person, but still experienced isolation, disconnection, and ultimately a less-than-satisfactory change in the music-making experience. Something crucial seemed to be lost from the original experience of choir, and the participants pointed towards human connection as that necessary ingredient that was still not completely restored in masked and physically-distanced choir spaces. The loss of connection with the audience during performances may explain why participants found the rehearsal process meaningless or purposeless, a sentiment reflected in the experiences of other choral singers (Youngblood et al., 2021). These experiences described by participants and other musicians fit the description of occupational alienation—a term used to describe an experience of “disconnectedness, isolation, emptiness, lack of a sense of spirit, or a sense of meaninglessness” (Townsend & Wilcock, 2004, p. 80) when participating in activities deemed meaningless or purposeless (Wilcock, 2006).

The spiritual aspects of an activity are of particular concern to occupational therapists. Models like the Canadian Model of Occupational Performance and Engagement (Townsend & Polatajko, 2013) and the Occupational Therapy Practice Framework (American Occupational Therapy Association, 2020) include spirituality as a person component. In occupational therapy, spirituality is not synonymous with one's religious values. Rather, it is what motivates people to find meaning and happiness through doing (Kang, 2003), and its meaning is unique to each individual (Mthembu et al., 2015). For amateur choristers, choral singing has been identified to give meaning and purpose to life (Livesey et al., 2012). For the present participants, the spiritual aspects of choral singing were twofold—connection to others and to self. The connection formed with others when making music together was described by several participants as spiritual. Specifically, Joy elaborated on how the community element of choral singing contributes to the feeling of belonging. This reflects the findings of Theorell et al. (2020), which found that both professional and amateur choral musicians identified social connection as what they missed most as a result of disrupted music-making during the COVID-19 pandemic. Choral singing as a form of connecting to oneself was evidenced by participants describing it as a big part of one's identity (Jonathan), what kept them grounded (Rochelle), and an act of taking care of one's spirit (Madeline). For professional musicians, engaging in music-making reinforces their professional identity. Theorell et al. (2020) also noted that professional choral musicians reported missing the aesthetic components of music-making more than amateur choral musicians. Engaging in alternative forms of music-making, which may not allow the individual to experience the aesthetic components of music-making to the same degree during the pandemic may contribute to a diminished sense of self. Participants’ explanations of what engaging in choral singing meant to them indicates that the occupation of choral singing includes spiritual aspects, regardless of whether they used the term “spiritual” or not.

If a person derives meaning from their doing, then disruption of that doing contributes to the feeling of being disconnected from oneself. Furthermore, if choir fosters the formation of connection among choral singers, then its disruption results in feelings of disconnection from others. Therefore, the concept of spiritual deprivation is another way to characterize the experience of choral singing for the participants during COVID-19. Spiritual deprivation is defined as a lack of or loss of meaning, sense of being, becoming, centeredness, connectedness, and/or ability to transcend (Kang, 2003). When circumstantial and environmental forces lead to the prioritization of physical needs, psychological and spiritual needs become neglected (Kang, 2003). In the case of the COVID-19 pandemic, public health restrictions were the circumstantial factor that prevented gatherings such as choral singing in order to protect the physical health of the community. As a result, the implications this had on the psychological and spiritual needs of choral musicians were not immediately taken into consideration. Psychological and spiritual needs therefore constitute important considerations for high-level choral musicians returning to their meaningful occupation of music-making.

It is important to note that the experiences described in this paper are not entirely reflective of Canadian choral musicians who experience certain forms of marginalization. The members of the Canadian choral association which assisted with recruitment are predominantly white. Thus, those who responded to recruitment were not those of visible minorities. Western art music has historically been used as a tool of colonialism in Canada and remains today associated with whiteness and the upper classes (Vaugeois, 2014). Occupational therapists working with musicians who are Indigenous, Black, or people of color should be aware of the colonial history of art music in Canada, but the present findings cannot contribute to the understanding of these experiences. These participants also hold privilege in that they have the time and space to respond to recruitment emails and participate in the interview. Had participation been compensated, perhaps the sample would have been more inclusive to visible minorities.

### Implications for Occupational Therapy Practice

Understanding the changes in the experience of choral singing during the COVID-19 pandemic holds value for occupational therapists. Musicians’ health is currently trending towards a more strengths-based, person-centered, and holistic approach (Guptill, 2014; Trondalen, 2013, pp. 864–865). This means that when physical and psychological injuries arise for choral musicians post-pandemic, occupational therapists are well-positioned to be a part of a team to assist in the recovery process. Clearly, the findings of this study support that engaging in choral singing is meaningful to high-level choral musicians, and is therefore within the scope of occupational therapy practice. Occupational therapists can benefit from knowing which aspects of music-making have been impacted during the COVID-19 pandemic and what elements of this experience facilitated or hindered meaningful engagement. Understanding these factors provides a starting point for occupational therapists to know how to help musicians re-engage with their music-making. This includes examining the environment to identify an array of components from masks to government-level policies that support or hinder musicians’ ability to pursue their meaningful occupation (Kirsh, 2015). Occupational therapy's scope of practice uniquely positions the profession to understand the experience of musicians during the COVID-19 pandemic and assist in their recovery on individual and community levels.

### Limitations

There are several limitations to this study. Participant recruitment and interviews were completed virtually through online interfaces. Thus, study participation required technological literacy, access to a computer or smartphone, and internet connection. Additionally, participants were sampled from across Canada, in which public health COVID-19 restrictions differed between provinces. The study findings are not representative of the choral musician experience from a specific geographical area in Canada. Instead, results show a generalized view of the music-making experience during COVID-19 as expressed by choral musicians from several Canadian provinces. Moreover, recruitment was not focused on race or gender diversity. Participants were not directly asked about their positions in intersectional power structures such as race or gender. Therefore, the findings do not provide insight on how marginalization may have affected participants’ experiences.

### Directions for Future Research

Given the present research data, the authors identify directions for future research. Participants whose primary income source was not music performance spoke of their conscious choice not to pursue music full-time and expressed concern for others who rely on music-making for their income during the COVID-19 pandemic. Those participants did not experience the same extent of income instability or concern for their future careers as many professional musicians had in prior studies (Cohen & Ginsborg, 2021; Spiro et al., 2021). However, participants in the present and other research studies expressed concern for the sustainability and longevity of professional music careers. Even prior to the pandemic, performance-based work was considered unpredictable, with musicians relying on sources of income beyond music (Botstein, 2020; Comunian et al., 2011; Gee & Yeow, 2021; Neil, 2010). Crosby and McKenzie (2021) call for change at the governmental level, advocating for policy evaluations to benefit musicians. Advocating for policy change to enhance a community's ability to engage in meaningful occupations is within the scope of occupational therapy practice, and can be considered a “professional imperative” (Kirsh, 2015). Future research exploring the experience of musicians who choose to pursue or not pursue professional music, and developing ideas for how the profession can be better supported financially and socially, should be considered.

## Conclusion

The experience of music-making for high-level choral musicians significantly changed during the COVID-19 pandemic. When the nature in which individuals engage with their meaningful occupation becomes altered, as it did for high-level choral musicians navigating choral singing in the context of the COVID-19 pandemic, it may result in negative impacts to their wellbeing. It is important for occupational therapists to understand the factors that facilitated these changed experiences in order to best support high-level choral musicians in continuing to engage in music-making within the context of the COVID-19 pandemic.

## Key Messages

- Lockdowns, social distancing, masking, and the concern over the safety of singing led to experiences of anxiety, isolation, and disconnection among choral musicians. The gradual return to singing as it was before the COVID-19 pandemic has been an exhausting and disheartening experience that these choral musicians are still contending with. Despite the challenges, choral musicians persisted to engage in music-making during the pandemic as a way to help them and others cope.
- Choral musicians identified that their music-making throughout the COVID-19 pandemic solidified the meaning and importance of choral music. Questions about the role of music in participants’ lives led to answers that were inherently spiritual, such as being essential for identity, belonging, and taking care of their spirit.
- Occupational therapists are uniquely qualified to be involved in the return of choral musicians to their meaningful occupation. Occupational therapists can involve themselves on the level of policy and on the individual level regarding the return to choir.

## Supplemental Material

sj-docx-1-cjo-10.1177_00084174221145823 - Supplemental material for Losing Life's Sparkle: Experiences of Canadian Choral Musicians During the COVID-19 Pandemic Click here for additional data file. Supplemental material, sj-docx-1-cjo-10.1177_00084174221145823 for Losing Life's Sparkle: Experiences of Canadian Choral Musicians During the COVID-19 Pandemic by Mary Jo A. Lozano, Stephanie L. Churcher, Madison J. Kirchner and Teri M. Slade in Canadian Journal of Occupational Therapy

## References

[bibr1-00084174221145823] Alsved M. Matamis A. Bohlin R. Richter M. Bengtsson P.-E. Fraenkel C.-J. Medstrand P. Löndahl J. (2020). Exhaled respiratory particles during singing and talking. Aerosol Science and Technology, 54(11), 1245–1248. 10.1080/02786826.2020.1812502

[bibr2-00084174221145823] American Occupational Therapy Association (2020). Occupational therapy practice framework: Domain and process. American Journal of Occupational Therapy, 74(Suppl. 2), 1–87. 10.5014/ajot.2020.74S2001 34780625

[bibr3-00084174221145823] Bailey B. A. Davidson J. W. (2005). Effects of group singing and performance for marginalized and middle-class singers. Psychology of Music, 33(3), 269–303. 10.1177/0305735605053734

[bibr4-00084174221145823] Bonde L. O. Juel K. Ekholm O. (2018). Associations between music and health-related outcomes in adult non-musicians, amateur musicians and professional musicians: Results from a nationwide Danish study. Nordic Journal of Music Therapy, 27(4), 262–282. 10.1080/08098131.2018.1439086

[bibr5-00084174221145823] Botstein L. (2020). The future of music in America: The challenge of the COVID-19 pandemic. The Musical Quarterly, 102(4), 351–360. 10.1093/musqtl/gdaa007

[bibr6-00084174221145823] Cai C. J. Carney M. Zada N. Terry M. (2021). Breakdowns and breakthroughs: Observing musicians’ responses to the COVID-19 pandemic. CHI’21: Proceedings of the 2021 CHI Conference on Human Factors in Computing Systems, 1–13. 10.1145/3411764.3445192

[bibr7-00084174221145823] Clift S. Hancox G. Morrison I. Hess B. Kreutz G. Stewart D. (2010). Choral singing and psychological wellbeing: Quantitative and qualitative findings from English choirs in a cross-national survey. Journal of Applied Arts and Health, 1(1), 19–34. 10.1386/jaah.1.1.19/1

[bibr8-00084174221145823] Cohen S. Ginsborg J. (2021). The experiences of mid-career and seasoned orchestral musicians in the UK during the first COVID-19 lockdown. Frontiers in Psychology, 12, 1–16. 10.3389/fpsyg.2021.645967 PMC8062715 33897549

[bibr9-00084174221145823] Comunian R. Faggian A. Jewell S. (2011). Winning and losing in the creative industries: An analysis of creative graduates’ career opportunities across creative disciplines. Cultural Trends, 20(3–4), 291–308. 10.1080/09548963.2011.589710

[bibr10-00084174221145823] Crosby P. McKenzie J. (2021). Survey evidence on the impact of COVID-19 on Australian musicians and implications for policy. International Journal of Cultural Policy, 28(2), 166–186. 10.1080/10286632.2021.1916004

[bibr11-00084174221145823] Damsgaard J. B. Brinkmann S. (2021). Me and us: Cultivating presence and mental health through choir singing. Scandinavian Journal of Caring Sciences, 36(4), 1134–1142. 10.1111/scs.13078 PMC9790328 35338510

[bibr12-00084174221145823] Dingle G. A. Brander C. Ballantyne J. Baker F. A. (2013). ‘To be heard': The social and mental health benefits of choir singing for disadvantaged adults. Psychology of Music, 41(4), 405–421. 10.1177/0305735611430081

[bibr13-00084174221145823] Gee K. Yeow P. (2021). A hard day’s night: Building sustainable careers for musicians. Cultural Trends, 30(4), 338–354. 10.1080/09548963.2021.1941776

[bibr14-00084174221145823] Good N. Fedak K. M. Goble D. Keisling A. L’Orange C. Morton E. Phillips R. Tanner K. John Volckens J. (2021). Respiratory aerosol emissions from vocalization: Age and sex differences are explained by volume and exhaled CO2. Environmental Science & Technology Letters, 8(12), 1071–1076. 10.1021/acs.estlett.1c00760

[bibr15-00084174221145823] Guptill C. (2014). Musicians’ health: A developing role for occupational therapists. Occupational Therapy Now, 16(6), 29–31. https://www.researchgate.net/profile/Christine-Guptill/publication/289668036_Musicians’_health_A_developing_role_for_occupational_therapists/links/5994ab27aca272ec908800c0/Musicians-health-A-developing-role-for-occupational-therapists.pdf

[bibr16-00084174221145823] Hildebrandt H. Nübling M. Candia V. (2012). Increment of fatigue, depression and stage fright during the first year of high-level education in music students. Medical Problems of Performing Artists, 27(1), 43–48. 10.21091/mppa.2012.1008 22543322

[bibr17-00084174221145823] Isaac M. J. McBroom D. H. Nguyen S. A. Halstead L. A. (2017). Prevalence of hearing loss in teachers of singing and voice students. Journal of Voice: Official Journal of the Voice Foundation, 31(3), 379.e21–379.e32. 10.1016/j.jvoice.2016.10.003 27839986

[bibr18-00084174221145823] Kang C. (2003). A psychospiritual integration frame of reference for occupational therapy. Part 1: Conceptual foundations. Australian Occupational Therapy Journal, 50(2), 92–103. 10.1016/j.hkjot.2017.05.003

[bibr19-00084174221145823] Kirsh B. H. (2015). Transforming values into action: Advocacy as a professional imperative. Canadian Journal of Occupational Therapy, 82(4), 212–223. 10.1177/0008417415601395 26502016

[bibr20-00084174221145823] Li X. Zhao M. Dong X. Zhao Q. Zhang X. (2021). Irrational beliefs surrounding the diagnosis of breast cancer in young Chinese women: An observational study. Medicine, 100(9), e25024. 10.1097/MD.0000000000025024 33655973 PMC7939144

[bibr21-00084174221145823] Livesey L. Morrison I. Clift S. Camic P. (2012). Benefits of choral singing for social and mental wellbeing: Qualitative findings from a cross-national survey of choir members. Journal of Public Mental Health, 11(1), 10–26. 10.1108/1746572121120727

[bibr22-00084174221145823] McBroom D. (2017). Empowering musicians: Teaching, performing, living: Why should I care about vocal health? Music Teachers National Association, 67(2), 32–35. https://www.jstor.org/stable/26385833

[bibr23-00084174221145823] McKie R. (2020, May 17). Did singing together spread coronavirus to four choirs? *The Guardian.* https://www.theguardian.com/world/2020/may/17/did-singing-together-spread-coronavirus-to-four-choirs

[bibr24-00084174221145823] McKinnon-Howe L. Dowdall J. (2018). Identifying knowledge gaps in clinicians who evaluate and treat vocal performing artists in college health settings. Journal of Voice, 32(3), 385.e7–385.e15. 10.1016/j.jvoice.2017.05.021 28760382

[bibr25-00084174221145823] Messick K. J. (2021). Music industry in crisis: The impact of a novel coronavirus on touring metal bands, promoters, and venues. In Bozkurt V. Dawes G. Gulerce H. Westenbroek P. (Eds.), The societal impacts of COVID-19: A transnational perspective (pp. 83–98). Istanbul University Press. 10.26650/B/SS49.2021.006.07

[bibr26-00084174221145823] Mthembu T. G. Ahmed F. Nkuna T. Yaca K. (2015). Occupational therapy students’ perceptions of spirituality in training. Journal of Religion and Health, 54(6), 2178–2197. 10.1007/s10943-014-9955-7 25294793

[bibr27-00084174221145823] Neil G. (2010). Status of the artist in Canada: An update on the 30th anniversary of the UNESCO Recommendation Concerning the Status of the Artist [White Paper]. Canadian Conference of the arts. http://www.ecthree.org/wp-content/uploads/2019/06/statusoftheartistreport1126101-copy.pdf.

[bibr28-00084174221145823] Pérez-Aldeguer S. Leganés E. N. (2014). Differences in psychological well-being between choristers and non-choristers in older adults. International Journal of Community Music, 7(3), 397–407. 10.1386/ijcm.7.3.397_1

[bibr29-00084174221145823] Perkins R. Mason-Bertrand A. Fancourt D. Baxter L. Williamson A. (2020). How participatory music engagement supports mental well-being: A meta-ethnography. Qualitative Health Research, 30(12), 1924–1940. 10.1177/1049732320944142 32755294 PMC7502980

[bibr30-00084174221145823] Primov-Fever A. Roziner I. Amir O. (2020). Songbirds must sing: How artistic voice users perceive their voice in times of COVID-19. Journal of Voice, 36(4), 586.e1–586.e5*,* in press. 10.1016/j.jvoice.2020.07.030 PMC7434435 32826119

[bibr31-00084174221145823] Rohrer K. (2017). Empowering musicians: Teaching, performing, living: When your body is your instrument–advocating for yourself and your life as a singer. Music Teachers National Association, 66(6), 54–57. https://www.jstor.org/stable/26385807

[bibr32-00084174221145823] Salleh A. (2021, November 26). What are the COVID-related risks of choir singing? Is it safe to go back? ABC News. https://www.abc.net.au/news/health/2021-11-27/covid-19-singing-choir-risk-safety-community/100630780.

[bibr33-00084174221145823] Shalanski L. Ewashen C. (2019). An interpretive phenomenological study of recovering from mental illness: Teenage girls’ portrayals of resilience. International Journal of Mental Health Nursing, 28(2), 492–500. 10.1111/inm.12552 30353956

[bibr34-00084174221145823] Sloan A. Bowe B. (2014). Phenomenology and hermeneutic phenomenology: The philosophy, the methodologies and using hermeneutic phenomenology to investigate lecturers’ experiences of curriculum design. Quality & Quantity, 48(3), 1291–1303. 10.1007/s11135-013-9835-3

[bibr35-00084174221145823] Spiro N. Perkins R. Kaye S. Tymoszuk U. Mason-Bertrand A. Cossette I. Glasser S. Williamon A. (2021). The effects of COVID-19 lockdown on working patterns, income, and wellbeing among performing arts professionals in the United Kingdom (April-June 2020). Frontiers in Psychology, 11, 594086. 10.3389/fpsyg.2020.594086 33643111 PMC7902701

[bibr36-00084174221145823] Stockman T. Zhu S. Kumar A. Wang L. Patel S. Weaver J. Spede M. Milton D. K. Hertzberg J. Toohey D. Vance M. Srebric J. Miller S. L. (2021). Measurements and simulations of aerosols released while singing and playing wind instruments. ACS Environmental Au, 1(1), 71–84. 10.1021/acsenvironau.1c00007 37155479 PMC8525345

[bibr37-00084174221145823] Grunwald Associates & Chorus America. (2019). The chorus impact study: Singing for a lifetime. https://www.arts.gov/sites/default/files/Research-Art-Works-ChorusAmerica.pdf

[bibr38-00084174221145823] Theorell T. Kowalski J. Theorell A. M. L. Horwitz E. B. (2020). Choir singers without rehearsals and concerts? A questionnaire study on perceived losses from restricting choral singing during the Covid-19 pandemic. Journal of Voice, 37(1), 146.e19–146.e27. 10.1016/j.jvoice.2020.11.006 33288380

[bibr39-00084174221145823] Thorne S. (2016). Interpretive Description : Qualitative Research for Applied Practice (Second edition). Routledge.

[bibr40-00084174221145823] Townsend E. Wilcock A. (2004). Occupational justice and client-centered practice: A dialogue in progress. Canadian Journal of Occupational Therapy, 71(2), 75–87. 10.1177/000841740407100203 15152723

[bibr41-00084174221145823] Townsend E. A. Polatajko H. J. (2013). Enabling Occupation II: Advancing an Occupational Therapy Vision for Health, Well-being, & Justice through Occupation (2nd ed.). CAOT-ACE.

[bibr42-00084174221145823] Trondalen G. (2013). Musicians. In Eyre L. (Ed.), Guidelines for music therapy practice in mental health (pp. 864–865). Barcelona Publishers.

[bibr43-00084174221145823] Turpin B. R. (2022). Voice Image: developing a new construct for vocal identity (Doctoral dissertation, The University of Western Ontario (Canada)).

[bibr44-00084174221145823] van Manen M. Adams C. A. (2010). Phenomenology. In Peterson P. Baker E. McGaw B. (Eds.), International encyclopedia of education (3rd ed., pp. 449–455). Elsevier. 10.1016/B978-0-08-044894-7.01539-6

[bibr45-00084174221145823] Vaugeois L. (2014). White Subjectivities, the Arts, and Power in Colonial Canada: Classical Music as White Property. In Stevens P. A. J. Dworkin A. G. (Eds.), The Palgrave Handbook of Race and Ethnic Inequalities in Education (pp. 45–67). Palgrave Macmillan. 10.1057/9781137317803.

[bibr46-00084174221145823] Whiteford G. (2000). Occupational deprivation: Global challenge in the new millennium. British Journal of Occupational Therapy, 63(5), 200–204. 10.1177/030802260006300503

[bibr47-00084174221145823] Wilcock A. A. (2006). An occupational perspective of health (2nd ed.). SLACK Incorporated. https://books.google.ca/books?id=voKCHG3xL70C&printsec=frontcover#v=onepage&q&f=false

[bibr48-00084174221145823] Youngblood F. K. Bosse J. Whitley C. T. (2021). How can I keep from singing? The effects of COVID-19 on the emotional wellbeing of community singers during the early stage lockdown in the United States. International Journal of Community Music, 14(2 + 3), 205–221. 10.1386/ijcm_00045_1

