# Supplementary material for: Losing Life's Sparkle: Experiences of Canadian Choral Musicians During the COVID-19 Pandemic
Source: Can J Occup Ther. 2023 Jan 22;90(2):125–35. doi: 10.1177/00084174221145823 (PMC9902788; doi:10.1177/00084174221145823)
Supplement: sj-docx-1-cjo-10.1177_00084174221145823 - Supplemental material for Losing Life's Sparkle: Experiences of Canadian Choral Musicians During the COVID-19 Pandemic [file sj-docx-1-cjo-10.1177_00084174221145823.docx]

Interview Guide

- Broad opener: Tell me about your work as a musician – what was your work as a musician like before the pandemic and now?
- Tell me about how you have engaged with music-making since the pandemic began.
  - How did you perform, practice, rehearse, etc.?
  - Have there been any positive aspects of the pandemic on your music-making?
  - Have there been any negative aspects of the pandemic on your music-making?
- What does meaningful music-making look like for you?
  - Probes:
    - Audience,
    - other ensemble members,
    - group atmosphere/belonging,
    - making money, communication,
    - spiritual component,
    - personal satisfaction,
    - rehearsal setting,
    - performance setting
- Have you been able to engage in meaningful music-making (as you define it) during the pandemic?
  - Are virtual concerts, small group rehearsals, etc, meaningful to you?
- What positive things (or lessons) will you take from this time into your future music-making post-pandemic?
